# Prevalence and incidence of cancer related lymphedema in low and middle-income countries: a systematic review and meta-analysis

**DOI:** 10.1186/s12885-020-07079-7

**Published:** 2020-06-29

**Authors:** Eric Torgbenu, Tim Luckett, Mark A. Buhagiar, Sungwon Chang, Jane L. Phillips

**Affiliations:** 1 Improving Palliative, Aged and Chronic Care through Clinical Research and Translation (IMPACCT), Faculty of Health, University of Technology Sydney, Sydney, New South Wales Australia; 2 Department of Physiotherapy and Rehabilitation Sciences, University of Health and Allied Sciences, Ho, Ghana; 3 Catholic Diocese of Parramatta, Parramatta, New South Wales Australia

**Keywords:** Lymphedema, Prevalence, Incidence, Risk factor, Cancer related lymphedema, LMICs

## Abstract

**Background:**

Little is known about the prevalence and incidence in low and middle-income countries (LMICs) of secondary lymphedema due to cancer. The purpose of the study is to estimate the prevalence and incidence in LMICs of secondary lymphedema related to cancer and/or its treatment(s) and identify risk factors.

**Method:**

A systematic review and meta-analysis was conducted. Medline, EMBASE and CINAHL were searched in June 2019 for peer-reviewed articles that assessed prevalence and/or incidence of cancer-related lymphedema in LMICs. Risk of bias was assessed using the Joanna Briggs Institute Critical Appraisal Checklist for Prevalence Studies. Estimates of pooled prevalence and incidence estimates were calculated with 95% confidence intervals (CI), with sub-group analyses grouping studies according to: country of origin, study design, risk of bias, setting, treatment, and lymphedema site and measurement. Heterogeneity was measured using *X*^2^ and I^2^, with interpretation guided by the Cochrane Handbook for Systematic Reviews.

**Results:**

Of 8766 articles, 36 were included. Most reported on arm lymphedema secondary to breast cancer treatment (*n* = 31), with the remainder reporting on leg lymphedema following gynecological cancer treatment (*n* = 5). Arm lymphedema was mostly measured by arm circumference (*n* = 16/31 studies), and leg lymphedema through self-report (*n* = 3/5 studies). Eight studies used more than one lymphedema measurement. Only two studies that measured prevalence of leg lymphedema could be included in a meta-analysis (pooled prevalence =10.0, 95% CI 7.0–13.0, *I*^*2*^ = 0%). The pooled prevalence of arm lymphedema was 27%, with considerable heterogeneity (95% CI 20.0–34.0, *I*^*2*^ = 94.69%, *n* = 13 studies). The pooled incidence for arm lymphedema was 21%, also with considerable heterogeneity (95% CI 15.0–26.0, *I*^*2*^ = 95.29%, *n* = 11 studies). There was evidence that higher body mass index (> 25) was associated with increased risk of arm lymphedema (OR: 1.98, 95% CI 1.45–2.70, *I*^*2*^ = 84.0%, *P <* 0.0001, *n* = 4 studies).

**Conclusion:**

Better understanding the factors that contribute to variability in cancer-related arm lymphedema in LMICs is an important first step to developing targeted interventions to improve quality of life. Standardising measurement of lymphedema globally and better reporting would enable comparison within the context of information about cancer treatments and lymphedema care.

## Background

Lymphedema is a distressing and often persistent condition that occurs when fluid accumulates in the extracellular tissue spaces causing swelling, predominately in the extremities [1]. Lymphedema is classified as congenital, primary or secondary. Secondary lymphedema occurs as a sequelae to another condition, such as the surgical and/or radiation treatments of cancer [2, 3].

Lymphedema is characterised by heaviness and discomfort, decreased range of motion, recurrent skin infections, elephantiasis verruca nostra, recurrent skin ulcers, cutaneous angiosarcoma, as well as psychological effects including depression, anxiety, and negative body image [4]. These effects impact adversely on quality of life [5].

Systematic reviews of the estimates incidence and prevalence of cancer-related lymphedema have focused almost exclusively on high-income countries (HICs). A 2013 systematic review and meta-analysis found the incidence of unilateral arm lymphedema post breast cancer treatment ranged from 8.4 to 21.4% [6]. Another systematic review estimated the prevalence of secondary lymphedema due to non-specific cancer in United Kingdom (UK) lymphedema specialist clinics (*n* = 11,555) to be 2.05–3.99:1000 [7]. Risk factors for lymphedema identified in the literature have included obesity at the time of a cancer diagnosis, receipt of chemotherapy, adjuvant radiation therapy, type of surgery, physiotherapeutic modalities, and number of lymph nodes removed [6, 8].

No review to date has reported on the pooled prevalence or incidence of lymphedema in LMICs and associated risk factors, making it difficult to advocate for and plan appropriate services to manage this condition.

### Aim

To estimate the prevalence and incidence in LMICs of secondary lymphedema related to cancer and/or its treatment(s) and identify risk factors.

## Methods

A systematic review and meta-analysis was registered with the International Prospective Register of Systematic Reviews (PROSPERO) [CRD42019137641] [9]. This review is reported in accordance with the preferred reporting items for systematic reviews and meta-analyses (PRISMA) guidelines [10]. This paper reports on the cancer-related lymphedema component of a larger review across lymphedema from all causes.

### Eligibility criteria

Primary studies in peer-reviewed journals of any design that estimated prevalence or incidence of secondary lymphedema in a sample from a LMIC, as defined by The World Bank Group [11] criteria. Where studies evaluated an intervention, only the baseline data were included. Studies using various measures of secondary lymphedema, including self-report and objective measures were included. Where studies were published in languages other than English, native language speakers were contacted to do the extraction according to the predefined criteria. Editorials, comment papers, review papers, case reports, and case series were excluded.

### Information sources

Database searches were conducted of Medline, Excerpta medica database **(**EMBASE) and Cumulative Index of Nursing and Allied Health Literature (CINAHL). A hand search of the reference lists of included studies was also performed.

### Search strategy

Databases were searched on seventh of June 2019 without any limit on date or language. Subject headings and keywords related to lymphedema and LMICs. The initial search strategy was developed in Medline and adapted for other bibliographic databases (refer to Table 1).

**Table 1:** Search strings for systematic review and meta-analysis

Medline	
No.	Searches
1.	(((((((((Afghanistan* or Benin* or Burkina Faso* or Burundi* or Central African Republic* or Chad* or Comoros* or Congo* or Eritrea* or Ethiopia* or Gambia* or Guinea-Bissau* or Haiti* or Korea Republic* or Liberia* or Madagascar* or Malawi* or Mali* or Mozambique* or Nepal* or Niger* or Rwanda* or Sierra Leone* or Somalia* or South Sudan* or Syrian Arab Republic* or Tajikistan* or Tanzania* or Togo* or Uganda* or Yemen* or Zimbabwe* or Angola* or Bangladesh* or Bhutan* or Bolivia* or Cabo Verde* or Cambodia* or Cameroon* or Congo* or Ivory Coast* or Djibouti* or Egypt* or El Salvador* or Georgia* or Ghana* or Honduras* or India* or Indonesia* or Kenya* or Kiribati* or Kosovo* or Kyrgyz Republic* or Lao PDP* or Lesotho* or Mauritania* or Micronesia* or Moldova* or Mongolia* or Morocco* or Myanmar* or Nicaragua* or Nigeria* or Pakistan* or Papua New Guinea* or Philippines* or Sao Tome) and Principe*) or Solomon Islands* or Sri Lanka* or Sudan* or Swaziland* or Timor-Leste* or Tunisia* or Ukraine* or Uzbekistan* or Vanuatu* or Vietnam* or West Bank) and Gaza*) or Zambia* or Albania* or Algeria* or American Samoa* or Armenia* or Azerbaijan* or Belarus* or Belize* or Bosnia) and Herzegovina*) or Botswana* or Brazil* or Bulgaria* or China* or Colombia* or Costa Rica* or Cuba* or Dominica* or Dominican Republic* or Equatorial Guinea* or Ecuador* or Fiji* or Gabon* or Grenada* or Guatemala* or Guyana* or Iran* or Iraq* or Jamaica* or Jordan* or Kazakhstan* or Lebanon* or Libya* or Macedonia* or Malaysia* or Maldives* or Marshall Islands* or Mauritius* or Mexico* or Montenegro* or Namibia* or Nauru* or Paraguay* or Peru* or Romania* or Russian Federation* or Samoa* or Serbia* or South Africa* or Saint Lucia* or Saint Vincent) and the Grenadines*) or Suriname* or Thailand* or Tonga* or Turkey* or Turkmenistan* or Tuvalu* or Venezuela*).mp.
2.	((Developing or underdeveloped or under-developed or less-developed or least-developed) adj world).mp.
3.	(Asia* or Africa* or Caribbean* or central America* or south America* or Melanesia* or Micronesia* or Polynesia*).mp.
4.	((developing or underdeveloped or under-developed or less-developed or least developed or less-economically developed or less-affluent or least-affluent) adj (country or countries or nation or nations or region or regions or economy or economies)).mp.
5.	(Third-world* or third world* or 3rd-world*).mp.
6.	Developing countries/ or exp. africa/ or exp. Caribbean region/ or exp. central America/ or latin America/ or exp. south america/ or asia/ or exp. asia, central/ or exp. asia, southeastern/ or exp. asia, western/ or exp. indian ocean islands/ or pacific islands/ or exp. melanesia/ or exp. micronesia/ or exp. west indies/
7.	or/1–6
8.	edema.mp. or Edema/
9.	oedema.mp.
10	Elephantiasis, Filarial/ or lymphoedema.mp. or Elephantiasis/
11.	exp Lymphedema/
12.	lymhoedema.mp.
13.	Breast Cancer Lymphedema/ or lymphedema.mp. or Non-Filarial Lymphedema/
14.	*lymphedema/
15.	*edema/
16.	exp Edema/
17.	8 or 9 or 10 or 11 or 12 or 13 or 14 or 15 or 16
18.	7 and 17
19	limit 18 to humans
20.	(((((((((‘Andorra’ or ‘Antigua) and Barbuda’) or ‘Argentina’ or ‘Aruba’ or ‘Australia’ or ‘Austria’ or ‘Bahamas’ or ‘Bahrain’ or ‘Barbados’ or ‘Belgium’ or ‘Bermuda’ or ‘British virgin islands’ or ‘Brunei Darussalam’ or ‘Canada’ or ‘Cayman Islands’ or ‘Channel Islands’ or ‘Chile’ or ‘Croatia’ or ‘Curacao’ or ‘Cyprus’ or ‘Czech Republic’ or ‘Denmark’ or ‘Estonia’ or ‘Faroe Islands’ or ‘Finland’ or ‘France’ or ‘French Polynesia’ or ‘Germany’ or ‘Gibraltar’ or ‘Greece’ or ‘Greenland’ or ‘Guam’ or ‘Hong Kong Sar’ or ‘China’ or ‘Hungary’ or ‘Iceland’ or ‘Ireland’ or ‘Isle of Man’ or ‘Israel’ or ‘Italy’ or ‘Japan’ or ‘Korea’ or ‘Kuwait’ or ‘Latvia’ or ‘Liechtenstein’ or ‘Lithuania’ or ‘Luxembourg’ or ‘Macao Sar’ or ‘Malta’ or ‘Monaco’ or ‘Netherlands’ or ‘New Caledonia’ or ‘New Zealand’ or ‘Northern Mariana Islands’ or ‘Norway’ or ‘Oman’ or ‘Palau’ or ‘Panama’ or ‘Poland’ or ‘Portugal’ or ‘Puerto Rico’ or ‘Qatar’ or ‘San Marino’ or ‘Saudi Arabia’ or ‘Seychelles’ or ‘Singapore’ or ‘Sint Maarten’ or ‘Slovak Republic’ or ‘Slovenia’ or ‘Spain’ or ‘Saint Kitts) and Nevis’) or ‘Saint Martin’ or ‘Sweden’ or ‘Switzerland’ or ‘Taiwan’ or ‘Trinidad) and Tobago’) or ‘Turks) and Caicos Islands’) or ‘United Arab Emirates’ or ‘United Kingdom’ or ‘United States’ or ‘Uruguay’ or ‘Virgin Islands’).mp.
21.	19 not 20

### Study selection

The first author (E.T.) assessed titles and abstracts of all citations retrieved by the search for relevance against the inclusion criteria, obtaining full texts as required to make a decision. 10% of articles were independently screened by a second author (T.L., M.B. or J.P.), with screening continued by E.T. alone after finding 100% agreement.

### Data extraction

Data were extracted by the first author (E.T.), with random checks performed by a second (T.L.). Data items extracted included: year and country, setting, aims, study design, sample size, sampling method, lymphedema site, stage, severity and duration, the type of management reported, and estimates of lymphedema prevalence or incidence.

### Risk of bias (quality) assessment

The first author (E.T.) independently assessed risk of bias for each study using the Joanna Briggs Institute Critical Appraisal Checklist for Prevalence Studies [12]. 20% of articles were independently assessed by the second author (T.L.), with the remaining risk of bias assessment continued by E.T. alone after a 100% agreement. Disagreements were resolved by discussion or, and when necessary, a third person arbitrating. The tool consists of 9 items which assess the internal and external validity of studies included in the quantitative analysis [12]. Studies were classified into low or high risk of bias using a cut-off of 70%.

### Statistical analysis

Meta-analyses of incidence and prevalence data were undertaken separately in accordance with the Cochrane Handbook for Systematic Reviews, using a random effects models [13]. The summary measure was the prevalent or incident percentage of people with lymphedema, with 95% confidence intervals. Following Ressing et al. [14], we assumed that cohort studies yielded estimates of incidence whereas cross-sectional studies yielded estimates of prevalence. Heterogeneity between estimates was measured using *X*^2^ and I^2^ statistics, using recommended thresholds [15]. For studies that used multiple lymphedema measurements, we prioritized the following measures based on level of objectivity [16, 17]: 1) circumferential measurement [18]; 2) perimetry (assessing difference in limb sizes, similar to the circumferential measurement) [6]; 3) limb volume measurement; 4) bioimpedence spectroscopy; or 5) self-report.

### Analysis of subgroups or subsets

Subgroup analyses were conducted on an a priori basis for studies classified according to whether or not estimation of prevalence/incidence was a stated aim of the study, and low risk of bias. Further subgroup analyses were conducted post hoc to explore any significant heterogeneity based on study characteristics such as country, setting, sample size, site and measurement of lymphedema and study design. Where studies were not considered sufficiently similar to be included in a meta-analysis, synthesis used a narrative approach based on the methods published by the Lancaster University, UK [19].

## Results

**Fig. 1:**
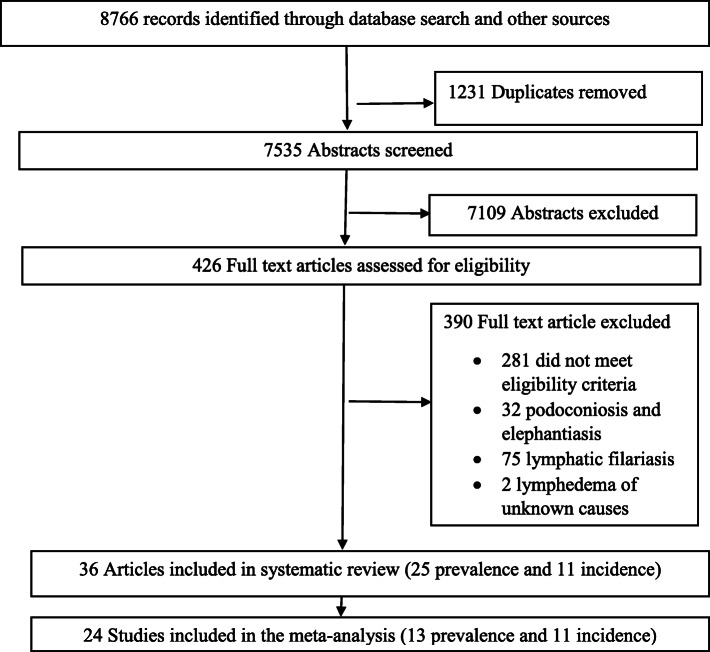
Flow diagram of study selection for inclusion in this review and meta-analysis

Of the 8766 articles that were retrieved, 1231 articles were excluded due to duplication. The remaining 7535 articles were evaluated, and 7109 were excluded based on their title and abstract. Next, 426 full-text articles were assessed and 389 were excluded, leaving 36 articles for inclusion reporting 36 studies (Refer to Fig. 1).

### Characteristics of included studies

The majority of studies (*n* = 34) focused on women (*n* = 12,145), while two studies [20, 21] involved both men and women. All studies were conducted between 2001 and 2019 and three studies [22–24] were reported in non-English language publications (Refer to Table 2).

**Table 2:** Studies reporting lymphedema prevalence or incidence

	Sample size	Stage of Diagnosis	Treatment received	Measurement method	Lymphedema definition	Prevalence or Incidence	Risk factors	Quality of Article
**Breast Cancer**								
(Yılmaz and Coşkun 2019) [23], Turkey	*N* = 64	Stage I-III	Breast cancer surgery	Self-reported and arm circumference	> 1.5 cm difference and lymphedema severity was defined as mild (if the difference between the extremities measurement is less than 3 cm), moderate (3–5 cm) and severe (> 5 cm)	14/64 (21.9%)	BMI and hand dominance	High risk
(Kibar, Dalyan Aras et al. 2017) [25], Turkey	*N* = 287	…	Modified Radical Mastectomy or Lumpectomy; Chemotherapy and/or Radiotherapy	Arm circumference	≥2 cm difference	119/287 (41.3%)	Axillary radiotherapy and ALND	Low risk
(Erdogan Iyigun, Selamoglu et al. 2015) [26], Turkey	*N* = 37	Stages 0-II	Surgery	Bioimpedance, clinical diagnosis, arm circumference	Impedance ratio > 10	8/37 (21.1%)	Age, surgical procedure, tumor localization, systemic treatment, body mass index, and lymphedema	High risk
(KİBar, Aras et al. 2015) [27], Turkey	*N* = 190	…	Level III ALND subsequent to a modified radical mastectomy or lumpectomy together with chemotherapy or radiotherapy	Arm circumference	≥2 cm difference	79/190 (41.5%)	Age, BMI, chemotherapy	High risk
(Ay, Kutun et al. 2014) [28], Turkey	*N* = 5064	Stage I & II	Mastectomy	Arm circumference	> 5 cm difference	1008/5064 (19.9%)	Employment status, Age, BMI, post-operative chemotherapy treatment. Post axillary radiotherapy was not significant	Low risk
(Ozcinar, Guler et al. 2012) [29], Turkey	*N* = 221	Stage I, II, cT1, 2 N0	Surgery	Arm circumference	> 2 cm difference	16/221 (7.2%)	Type of the surgical procedure done, RT to regional lymphatics, ALND and RT Administration to axilla	Low risk
(Ozaslan and Kuru 2004) [30], Turkey	*N* = 240	Stage I-III	Surgery	Arm circumference	> 4 cm difference	68/240 (28%)	Axillary radiotherapy; BMI	Low risk
(Rebegea, Firescu et al. 2015) [21], Romania	*N* = 305	Stages I-IV	Surgery	Arm circumference	≥2 cm difference	18/305 (5.9%)	number of lymph nodes removed; stage of the disease; chemotherapy and hormonal therapy	Low risk
(Borman, Yaman et al. 2018) [51], Brazil	*N* = 135	Post breast cancer related with no advanced malignancy	…	Volume measurement	> 10% volume difference	125/135 (92.5%)	…	High risk
(Vieira, Silva et al. 2018) [32], Brazil	N = 16	ECOG scores of 0 to 1	Radiotherapy	Arm circumference	≥200 mm difference	4/16 (25%)	…	High risk
(Borman, Yaman et al. 2017) [51], Brazil	*N* = 188	Subclinical, reversible, spontaneous irreversible, elephantiasis and stages I-III with mean time past after the surgery was 21.5 ± 27.5 months	Surgery	Self-reported arm swelling, arm circumferences	A positive Stemmer’s sign	170/188 (90%)	Lymphedema awareness	High risk
(Godoy, Dias et al. 2014) [33], Brazil	*N* = 1583	…	Surgery	Self-reported arm LE	Swelling of the arm	12/1583 (0.8%) suffered LE; 12/32 (37.5%) suffered LE due to axillary dissection	…	High risk
(Paiva, Rodrigues et al. 2013) [34], Brazil	*N* = 250	…	Surgery (more than 6 months)	Perimetry	≥2 cm difference	112/250 (44.8%)	ALND; SLNB; time after surgery	Low risk
(do Nascimento, de Oliveira et al. 2012) [24], Brazil	*N* = 707	Stages I-IV (presented with overweight, diabetic, hypertensive and shoulder dysfunction)	Surgery	Self-reported LE and perimetry	Swelling of the arm	164/707 (23.2%)	…	High risk
(de Godoy, Barufi et al. 2012) [35], Brazil	*N* = 35	…	Breast cancer treatment	Self-reported presence of chest swelling; Bioimpedance	> 100 g difference	4/35 (11.42%)	…	High risk
(Campanholi, Duprat et al. 2011) [20], Brazil	*N* = 84	…	Surgery	Arm and leg circumference; volume measurement; self-reported	> 10% difference in volume; 0–10% = normal, 10.1–20% = mild, 20.1–40% = moderate, 40.1–80% = marked, > 80.1% = severe in the arms and classified in the leg as 0–6.5% = normal, 6.6–20% = mild, 20.1–40% = moderate and > 40.1% = severe	7/40 (17.5%) in the arm; 26/44 (59.1%) in the lower limb	Local lymphadenecto-my including axillary, inguinal and ilioinguinal	High risk
(Bergmann, Bourrus et al. 2011) [36], Brazil	*N* = 220	Stages IIA, IIB, IIIA and IIIB	Advanced Breast Cancer Treatment	Self-report, volumetric measurement	> 200 ml difference in volume	13/220 (6.6%)	Obstruction of lymphatic drainage and clinical stage of the condition; Radiotherapy and chemotherapy and delay in accessing neo-adjuvant therapy	Low risk
(Velloso, Barra et al. 2011) [37], Brazil	*N* = 45	…	Surgery (21.3 months)	Arm circumference	> 10% difference	2/45 (4.4%)	…	High risk
(Alem and Gurgel 2008) [38], Brazil	*N* = 29	Post-surgery with mean time for breast cancer 86.1 ± 81.6 months.	Breast Cancer Surgery	Arm circumference	≥2 cm difference; a restriction of 20° or more in flexion and/or abduction in ROM.	23/29 (79.0%)	…	High risk
(Paim, Lima et al. 2008) [5], Brazil	*N* = 96	…	Surgery	Arm circumference/ perimetry and Clinical diagnosis	> 1 cm and any two of lymphedema symptoms of limb heaviness, swelling, tightness or firmness	17/96 (17%) and the prevalence with treatment; ALND 14/48 (29.2%) and SLNB 2/48 (4.2%)	ALND; SLNB	Low risk
(Batiston and Santiago 2005) [39], Brazil	*N* = 160	Stage I - IV	Radical surgery (68.8%) and conservative surgery (31.2%)	Self-reported swelling	…	47/160 (29.2%)	Time after surgery to physiotherapy rehabilitation	High risk
(Elumelu-Kupoluyi, Adenipekun et al. 2013) [40], Nigeria	*N* = 63	Stage II	Radiotherapy	Clinical diagnosis	A positive stemmer’s sign	55/63 (78%) in the arm.	…	High risk
(Khanna, Gupta et al. 2019) [41], India	*N* = 98	Locally advanced (IIIB) and Early/palpable stage (I-IIIA)	Breast carcinoma treatment; Mastectomy and Wide local incision	Arm circumference	≥2 cm difference in limb between pre-op and post-op measurements	23/98 (23.5%)	Drainage of seroma, type of treatment especially axillary radiotherapy and skin necrosis, chemotherapy	Low risk
(Rastogi, Jain et al. 2018) [42], India	*N* = 100	Stages IIA, IIB, IIIA, IIIB and IIIC	Mastectomy, Radiotherapy and Axillary Lymph Node Dissection	Arm circumference	≥2 cm difference	13/100 (13.0%) and 13/33 (39.4%) recorded by patients with BMI > 25	BMI; Number of lymph nodes removed; regional lymph node radiated	High risk
(Gopal, Acharya et al. 2017) [43], India	*N* = 199	Early and locally advanced stages	Radiotherapy, Lymph Node dissection, Surgery and Chemotherapy	Arm circumference	> 5% difference	85/199 (42.7%)	stage of cancer, BMI, receiving radiotherapy or chemotherapy, number of lymph nodes removed	High risk
(Nandi, Mahata et al. 2014) [52], India	*N* = 135	Grades I-IV	Chemotherapy, Radiotherapy and Mastectomy	Self-reported	…	9/135 (6.7%)	…	High risk
(Raja, Damke et al. 2014) [44], India	N = 30	Stage I and II	Modified Radical Mastectomy with Axillary Clearance	Self-reported	Lymphedema grading system of mild, moderate and severe	17/30 (56.7%)	…	High risk
(Deo, Ray et al. 2004) [45], India	*N* = 299	Stage I, II & III	Post Breast cancer treatment (Surgery & Radiotherapy)	Arm Circumference	> 3 cm	100/299 (33%)	Axillary irradiation; comorbidities.	Low risk
(Halder, Morewya et al. 2001) [46], Papua New Guinea (East Asia)	*N* = 790	Stages I-IV	Lumpectomy and Mastectomy	Self-reported	…	3/790 (0.4%)	…	High risk
(Haddad, Farzin et al. 2010) [54], Iran	*N* = 355	Cases of no evidence of recurrence or metastases after surgery	Surgery	Arm circumference and self-reported swelling	> 10% difference	63/355 (17.5%)	Type of surgery, treatment with radiotherapy, and prescription of a supraclavicular field of radiation	Low risk
(Honarvar, Sayar et al. 2016) [47], Iran	*N* = 683	…	Modified radical mastectomy, conservative surgery, chemotherapy, radiotherapy and hormone therapy	Arm circumference	≥2 cm difference and a positive stemmer’s sign	400/683 (58.6%)	Type of surgery, treatment with radiotherapy, physical activity, modified radical mastectomy, BMI, hormone therapy, size of tumor, and number of excised or affected lymph nodes.	Low risk
(Morcos, Al Ahmad et al. 2013) [48], Jordan	*N* = 531	…	Surgery, chemotherapy and radiotherapy	Arm circumference	≥2 cm difference	114/531 (21.4%)	Surgery type received	High risk
**Vulvar Cancer**								
(de Melo Ferreira, de Figueiredo et al. 2012) [49], Brazil	N = 50	Stage I-IV	Vulvectomy	Clinical diagnosis, observation and palpation by the clinician	Severity and limb functions considered based on disabilities reported	28/56 (50%); 17/28 (60.7%) among cases and 3/28 (10.7%) among the control	Severity and BMI	High risk
(Eke, Alabi-Isama et al. 2010) [53], Nigeria	N = 11	Stages IB-IV	Vulvar carcinoma surgery	Self-reported LLE	…	1/11 (9.1%)	…	High risk
**Cervical Cancer**								
(Marin, Pleşca et al. 2014) [50], Romania	*N* = 324	…	Lymphadenohysterocolpectomy; Radical hysterectomy	Self-reported	…	37/324 (11.4%); lower limb lymphedema (13.5% III vs 11.5% II)	…	High risk
(Elumelu-Kupoluyi, Adenipekun et al. 2013) [40], Nigeria	N = 63	Stage II	Radiotherapy	Clinical diagnosis	A positive stemmer’s sign	8/63 (13%) in the leg	…	High risk
(Dem, Kasse et al. 2001), Senegal [22]	*N* = 86	Stages I-IV	Cervical cancer treatment	Self-reported	…	6/86 (6.98%) in the leg	…	High risk

While the majority of studies were conducted in Brazil (*n* = 12) and Turkey (*n* = 9), most other regions with LMIC were represented, including: South America (*n* = 12) [5, 20, 24, 32–39, 49], Europe (*n* = 11) [21, 23, 25–31, 50, 51], Southern Asia (*n* = 6) [41–45, 52], West Africa (*n* = 3) [22, 40, 53], Middle East (*n* = 3) [47, 48, 54] and East Asia and Pacific (*n* = 1) [46].

Most studies were cross-sectional (*n* = 21), with a smaller number of prospective cohort (*n* = 8), retrospective cohort (*n* = 3) and case-control studies (*n* = 4). The majority of studies (*n* = 34) reported exclusively on either arm (*n* = 30) or leg (*n* = 4) lymphedema, while two [20, 40] reported on both. One study reported on lymphedema of the chest and arm secondary to breast cancer treatment [35]. This study was the only study to use bioelectric impedance to diagnose lymphedema [35]. Other methods used for measuring and defining lymphedema included: tape measurement (*n* = 16) [21, 25, 27–30, 32, 37, 38, 41–43, 45, 47, 48, 54]; patient self-report (*n* = 8) [22, 33, 39, 44, 46, 50, 52, 53]; water volumeter (*n* = 2) [31, 36]; palpation and clinical diagnosis (*n* = 2) [40, 49]; and perometer (*n* = 1) [34].

Twenty-five studies reported lymphedema prevalence and 11 studies reported incidence.

Of the three studies that explored the risk of developing lymphedema associated with cancer staging both in the leg and arm, two involved women with breast cancer [30, 41] and the other, women with vulvar cancer [49]. Four studies reported on the risk of developing arm lymphedema associated with breast cancer treatment among women who had sentinel lymph node biopsy [5, 21, 29, 37]. Variations in the timing or the onset of cancer related lymphedema ranged from 3 months to over 5 years post diagnosis and treatment. The type of management received by women with cancer related lymphedema included: lymphatic drainage [29], physiotherapeutic modalities such as care for the affected limb, home exercises and self-lymphatic drainage [24, 28, 39], hormonal therapy [54] and neo-adjuvant therapy including radiotherapy and chemotherapy [34, 38].

### Synthesis

#### Arm lymphedema following breast cancer treatment

The majority of studies (*n* = 31) reported arm lymphedema secondary to breast cancer treatment. However, lymphedema was defined differently based on the method of measurement used. Half of these studies (*n* = 16) used circumferential measurements [21, 25, 27–30, 32, 37, 38, 41–43, 45, 47, 48, 54]. The remainder either used self-reports of swelling in the arms (*n* = 5) [33, 39, 44, 46, 52], volumetric measurement (*n* = 2) [31, 36], perimetry (*n* = 1) [34] and/or bioimpedance spectrometry (*n* = 1) [26]. Six studies used more than one method of arm lymphedema diagnosis [5, 20, 23, 24, 36, 51].

Eleven studies (*n* = 11) compared circumferential measurement in bilateral limbs using a range difference of ≥2 cm as indicative of lymphedema. One study from Brazil only used a difference of ≥1 cm circumferential measurement in the presence of any other two lymphedema symptoms of heaviness, swelling, tightness or firmness in the affected limb [5]. Another study [23] which examined the upper extremity disorders among breast cancer women undergoing surgery measured lymphedema as circumferential measurement difference ≥ 1.5 cm in the affected limb. There was only one large population study involving Turkish women with breast cancer (*n* = 5064), which used a cut-off difference of ≥5 cm in the affected limb as a diagnosis for lymphedema [28]. All Turkish studies (*n* = 7) measured arm lymphedema by the circumferential method, while the Brazilian (*n* = 3) [24, 33, 39] and Indian (*n* = 2) [44, 52] studies used patients’ self-reports.

Studies which used the volumetric measurement defined lymphedema to be a cut-off difference in volume based on circumferential measurements of both limbs > 10% percent [31, 36]. Lymphedema was diagnosed as an impedance ratio of greater than 10 in the affected limb using the bioimpedance spectrometer [26].

##### Prevalence of arm lymphedema following breast cancer treatment

The most common method of arm lymphedema measurement was arm circumference (*n* = 16), while several studies (*n* = 9) also used more than just one lymphedema measurement. One study used lymphoscintigraphy as a technique in the measurement of lymphedema among Brazilian post-breast cancer women [37]. All studies included in this review reported prevalence of arm lymphedema secondary to breast cancer treatment. Twenty-five studies reported prevalence estimates [5, 22, 23, 25, 27, 31–40, 43, 45–51, 53, 54]. The prevalence estimate among post breast cancer treated women varied from 0.4% in Papua New Guinea [46] to 92.5% reported by a Brazilian study [31]. The lowest estimate of 0.4% was reported by self-report of lymphedema [46]. Of the two studies that reported on sentinel lymph node biopsy, the prevalence estimates were relatively low compared with other studies; 4.4% (95% CI 1.0–15.0) [37] and 17.0% (95% CI 11.0–27.0) [5].

**Fig. 2:**
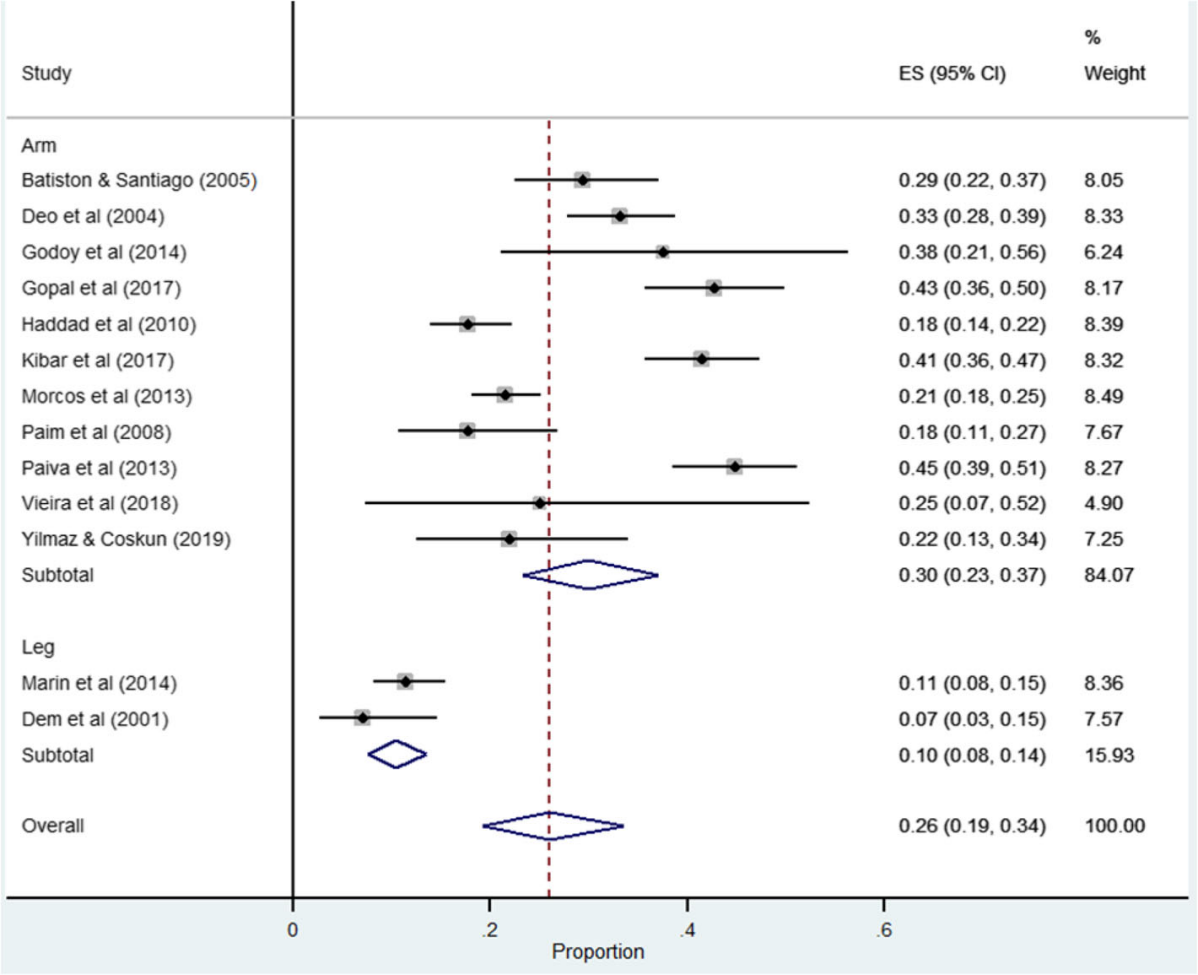
Forest plot of pooled prevalence of arm and leg lymphedema

**Fig. 3:**
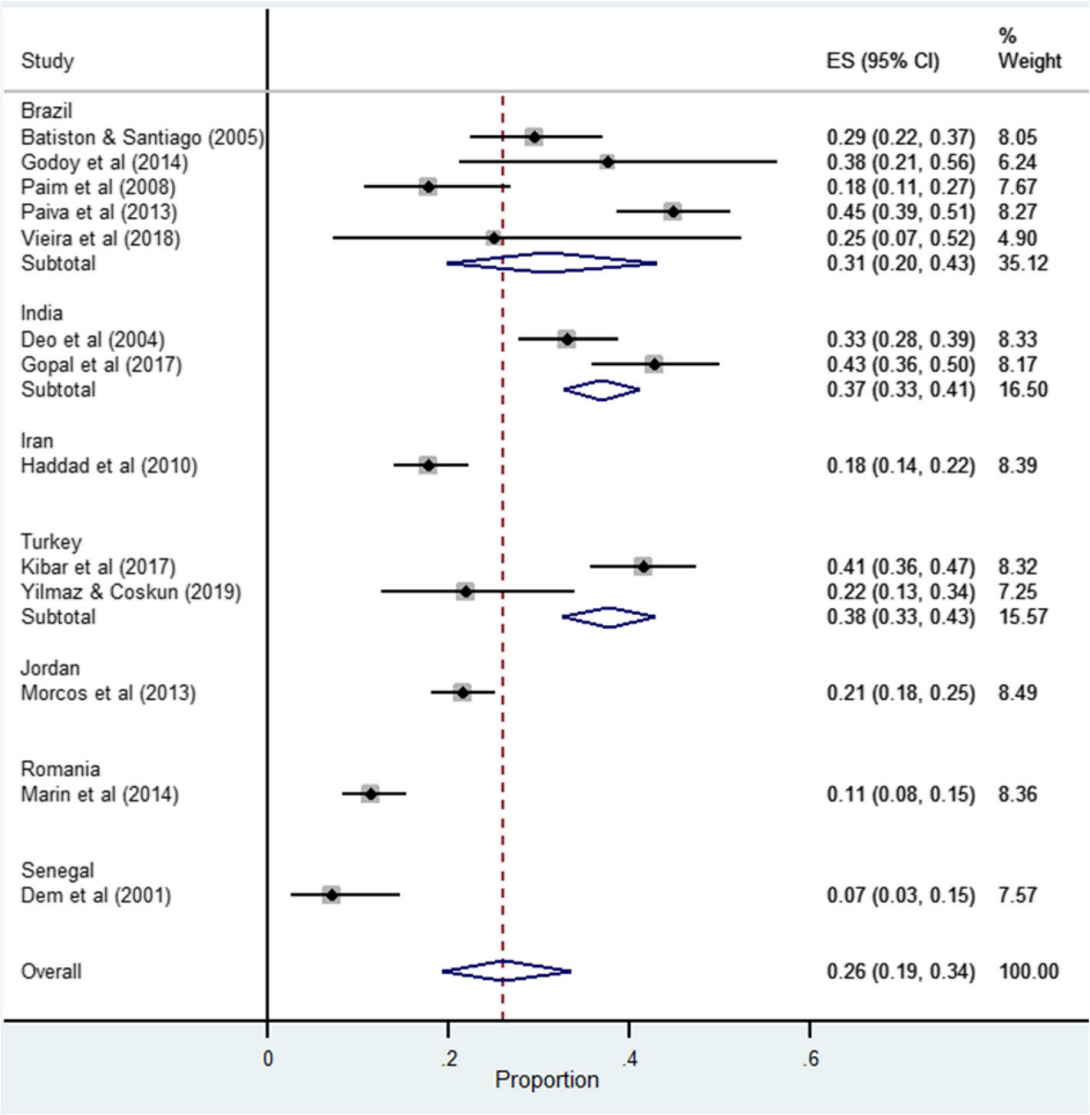
Forest plot of pooled prevalence of cancer related lymphedema based on country of study publication

Using data abstracted from 13 studies the pooled estimate for prevalence of breast cancer related lymphedema was 30% (95% CI 24–37). There was considerable heterogeneity among studies (I^2^ = 91.66%, *p* = 0.001) (refer to Fig. 2). Heterogeneity was not reduced in a subgroup analysis of studies grouped by a single country, Brazil (pooled prevalence = 31, 95% CI 19.0–43.0, *I*^2^ = 87.21%, *n* = 5 studies). Studies from the Middle East (i.e. Iran [54], Jordan [48] and Turkey [23]) demonstrated considerable heterogeneity (I^2^ = 94.69%, *p* = 0.001), which increased to considerable heterogeneity when a second Turkish study [25] was included (I^2^ = 99.67%, *p* = 0.001). The pooled prevalence recorded by the two Turkish studies was 37% (95% CI 32–42) among breast cancer women receiving treatment in cancer units (refer to Fig. 3).

##### Incidence of arm lymphedema following breast cancer treatment

Eleven [11] studies reported incidence of unilateral arm lymphedema [20, 21, 24, 26, 28–30, 41, 42, 44, 52], while one study reported lymphedema of both arm and leg [20]. The follow up periods varied among studies from 6 months to over 5 years post- cancer treatment.

**Fig. 4:**
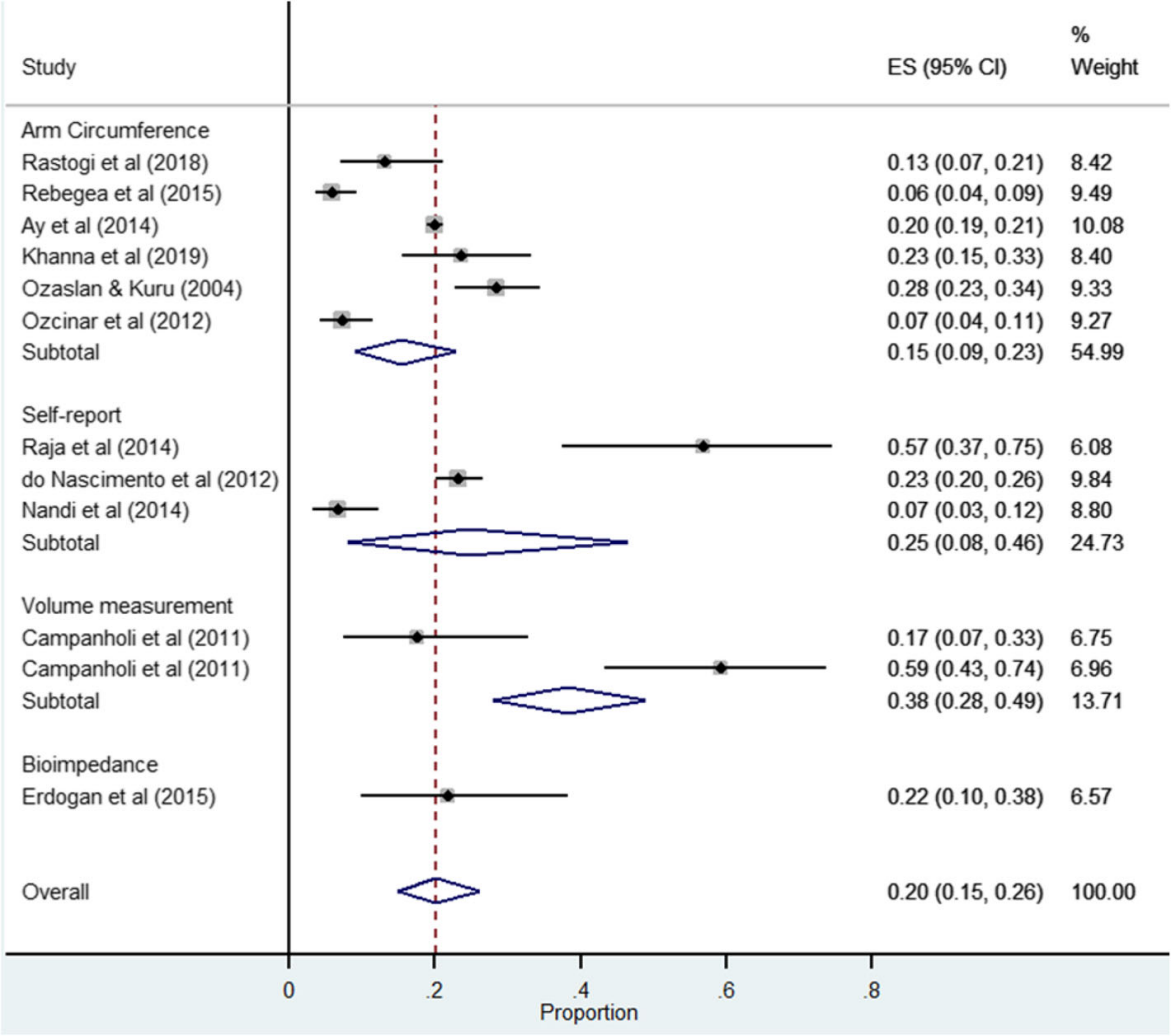
Forest plot of pooled lymphedema incidence according to arm lymphedema measurement methods

The lowest incidence was 5.9% after breast cancer treatment in Romania [21] with a mean follow up period of 24 months, who received sentinel lymph node biopsy. The highest incidence was 56.7% recorded in an Indian study with 6-month follow up after modified radical mastectomy treatment for breast cancer patients [44]. Breast cancer related lymphedema incidence in Turkey ranged from 7.2% recorded within a population sample with a median follow up of 64 months [29] to 28% in a population sample with a median follow up of 30 months after breast cancer treatment [30]. The incidence of arm lymphedema reported by the Brazilian studies ranged from 17.5 to 23.2% [20, 24]. The pooled incidence was 21% (95% CI 15.0–26.0, *I*^*2*^ = 95.29%, *n =* 11 studies) with considerable heterogeneity, while that reported by circumferential measurement was 16% (95% CI 9.0–23.0, *I*^*2*^ = 96.54%, *n* = 6 studies) (refer to Fig. 4). The estimated pooled incidence by all other methods of assessment was between 16.0% (circumferential measurement) and 26.0% (self-report).

##### Risk factors of lymphedema following breast cancer treatment

**Fig. 5:**
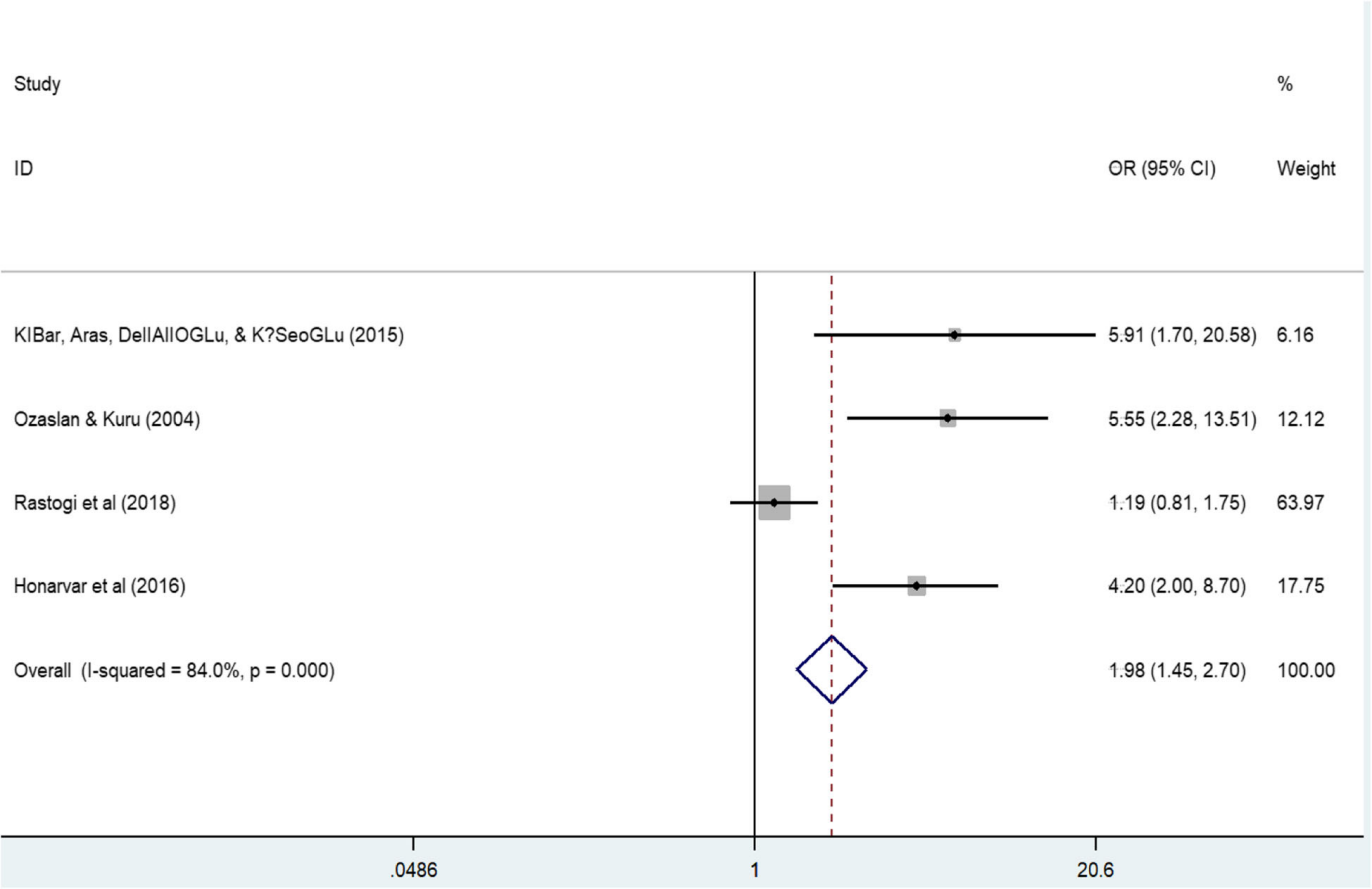
Effect of BMI on risk of arm lymphedema: adjusted effect measure and 95% confidence interval (CI) by study

Ten of the 11 studies reporting on lymphedema risk factors, focused on the risk of developing arm lymphedema following breast cancer treatment [21, 27–30, 34, 41, 42, 45, 47]. One study [28] reported that individuals with body mass index (BMI) of ≥30 were 6.64 times more likely to develop arm lymphedema than those with BMI ≤17.9. The risk of developing arm lymphedema among breast cancer women with BMI ≥25 ranges from the odds ratio (OR) of 1.5 to 5.9 compared to participants with BMI < 25 [27, 28, 30, 42, 47]. We obtained a pooled effect estimate OR of 1.98, 95% confidence interval (CI): 1.45 to 2.70 (*P* < 0.0001; *I*^2^ = 84.0%) in a random effect meta-analysis (refer to Fig. 5).

Axillary radiotherapy treatment is a significant risk with an OR ranging from 2.7 to 4.4 [21, 27, 29]. Four studies examined the risk of developing arm lymphedema associated with higher number of lymph node removal among breast cancer survivors [21, 27, 42, 47]. The removal of lymph nodes of > 25 during mastectomy was associated with a risk of developing arm lymphedema [4.88 (OR2.25–10.58)] among breast cancer woman compared with when less number of lymph nodes were removed [21]. Higher nodal ratio [1.135 (Hazard ratio (HR) 1.037–1.243)] was also found to be associated with higher risk of arm lymphedema [42]. Lumpectomy was not a significant risk factor for arm lymphedema [27].

Modified radical mastectomy was associated with an OR of 4.3 (95% CIs: 2.3–7.9) risk than those who did not and participants who received radiotherapy had an OR of 3.9 (95% CIs: 1.8–8.2) risk of developing arm lymphedema compared with those who did not [47]. The length of time after surgery for breast cancer was also reported to be 9.7 times higher among breast cancer women who had surgery more than 5 years as compared to those with less years [34].

Other risk factors identified to significantly affect lymphedema among breast cancer survivors include: past history of limb damage had an OR of 1.7 (95% CIs: 0.9–3.1) [47], presence of a co-morbid condition with a HR of 0.1593 (95% CIs: 1.1441–2.9369) [45], post radiotherapy moist desquamation had an OR of 4.34 (95% CIs: 1.07–17.65) [41], and presence of seroma after breast cancer surgery [34]. Women with breast cancer tumour invasion were 13.7 times at risk of developing arm lymphedema compared to those women who did not receive tumour invasion [47]. Cancer stage was not significant in arm lymphedema following breast cancer treatment [34, 41] (refer to Table 3).

**Table 3:** Risk factors of lymphedema

Risk factor	Author	Risk ratio/Hazard ratio	Stage and Measurement
**1. Arm Lymphedema**			
BMI > 25 Age > 60 Number of metastatic LNs Having a Breast/Chest-wall RT Having Axillary RT Lumpectomy^a^	(KİBar, Aras et al. 2015) [27]	5.911 (OR1.698–20.583) 3.680 (OR1.076–12.583) 1.115 (OR1.043–1.192) 3.249 (OR1.742–6.060) 4.375 (OR1.439–13.306) 0.294 (OR0.062–1.402)^a^	Patients undergoing Level III Mastectomy/ Lumpectomy/ Chemotherapy/ Radiotherapy Arm circumference measurement
Having mastectomy (ALND) + RT	(Ozcinar, Guler et al. 2012) [29]	Patients with ALND + RT had statistically increased rate of lymphedema than patients with ALND and without RT (*p* = 0.030)	Stage I, II who underwent mastectomy Arm circumference measurement
BMI > 25 Axillary Radiotherapy Stage of the cancer (I-III)	(Ozaslan and Kuru 2004) [30]	5.55 (RR2.28–13.51) 2.75 (RR1.48–5.08) Not significant	Stage I-III Arm circumference measurement
Number of lymph node removed 16–25 Removal > 25 Adjuvant RT + LND Chemotherapy	(Rebegea, Firescu et al. 2015) [21]	1.85 (OR1.27–2.71) 4.88 (OR2.25–10.58) 3.87 (OR1.39–6.51) 1.45 (OR1.12–2.24)	Stages I-IV Arm circumference measurement
Presence of seroma after breast cancer surgery Staging of cancer^a^ Time after surgery	(Paiva, Rodrigues et al. 2013) [34]	2.71(PR1.49–4.91) 1.15(PR0.78–2.92)^a^ Surgery for > 5 years is 9.7 times higher frequency than < 5 years	Women undergoing oncology follow up Perimetry
Staging (Locally advanced III)^a^ Post RT skin necrosis	(Khanna, Gupta et al. 2019) [41]	2.21(OR 0.54–9.04)^a^ 4.34 (OR1.07–17.65)	Early and locally advanced stages Arm circumference measurement
Higher BMI Increasing number of lymph nodes dissected Higher nodal ratio Regional Lymph Node Radiation (RLNR)	(Rastogi, Jain et al. 2018) [42]	1.191 (HR0.809–1.755) 1.445 (HR1.116–1.872) 1.135 (HR1.037–1.243) 1.020 (HR0.042–24.571)	Stage II – III Arm circumference measurement
Axillary RT Presence of co-morbid condition	(Deo, Ray et al. 2004) [45]	0.0709 (HR2.3222–7.1601) 0.1593 (HR1.1441–2.9369)	Stage I-III Arm circumference measurement
Engaging in moderate to severe physical activity BMI of ≥25 Invasiveness of the tumor Modified Radical Mastectomy Having radiotherapy Past history of limb damage Number of lymph nodes removed	(Honarvar, Sayar et al. 2016) [47]	14.0 (OR2.6–73.3) 4.2 (OR2.0–8.7) 13.7 (OR7.3–25.6) 4.3 (OR2.3–7.9) 3.9 (OR1.8–8.2) 1.7 (OR0.9–3.1) 1.1 (OR1.0–1.1)	Women with breast cancer Arm circumference measurement
BMI	(Ay, Kutun et al. 2014) [28]	BMI of 25–29.9 was 1.445 times more likely to develop lymphoedema than a patient with a BMI of < 17.9 (p < 0.001), and a patient with a BMI of 30–34.9 was 6.643 times more likely to develop it than a patient with a BMI of < 17.9 (*p* < 0.001).	Stage I & II Arm circumference measurement
**2. Leg Lymphedema**			
Age BMI Staging^a^	(de Melo Ferreira, de Figueiredo et al. 2012) [49]	1.09 (OR1.00–1.18) 1.34 (OR1.01–1.77) 0.33 (OR0.02–5.33)^a^	Stage I-IV Clinical diagnosis

^a^Not significant in the final model

*RT* Radiotherapy, *LN* Lymph node, *BMI* Body mass index; Lymph node dissection

#### Leg lymphedema following gynecological cancer treatment

All five studies that reported leg lymphedema used either patient self-report (*n* = 3) or palpation or clinical diagnosis (*n* = 2). Studies which used the self-report method of lymphedema diagnosis only used either palpation or observation methods of identifying lymphedema in the affected limbs of the patients [22, 50, 53]. These were based on patients’ reports of swelling in the legs alone. In the case of the clinical diagnosis, lymphedema was identified as present when a positive Stemmer’s sign was recorded [40].

##### Prevalence of leg lymphedema following gynecological cancer treatment

Of the five studies reporting on leg lymphedema, three focused on the prevalence of leg lymphedema secondary to cervical cancer treatment; two West African and one Romanian [22, 40, 50]. The prevalence estimates were similar; 7.0% (95% CI 3–15) [22], 11.0% (95% CI 8–15) [50] and 13% (95% CI 7–23) [40]. The three studies [22, 40, 50] that reported on leg lymphedema following cervical cancer reported a pooled prevalence of 10% (95% CI 7–13) with considerable heterogeneity. The method of measurement of lymphedema was self-report and none of these studies explored leg lymphedema risk factors.

Two studies reported leg lymphedema prevalence based on clinical diagnosis among women who received vulvectomy [49, 53]. The prevalence varied widely from 60.1% in the Brazilian study to 9.1% in the Nigerian study [53].

The incidence of leg lymphedema was reported in only one study, which focused on patients following inguinal and ilioinguinal lymphadenectomies in Brazil and identified an incidence of 59.1% [20].

##### Risk factors of lymphedema following vulvar cancer treatment

One study reported the risk of developing leg lymphedema following vulvar cancer treatment [49]. The risk of leg lymphedema following vulvar cancer included age associated with an OR of 1.09 (95% CIs: 1.00–1.18) and a BMI with an OR of 1.34 (1.01–1.77) [49] (refer to Table 3).

##### Sub-group analyses

Planned sub-group analyses failed to significantly reduce heterogeneity. Heterogeneity based on: country of study publication and the type of cancer was 95.29%; study region was 93.85%; sample size, the type of measurement of lymphedema, and the design of the study were 94.69%. The level of heterogeneity was 97.2% (*n* = 5 studies) for incidence and 94.89% (*n* = 6 studies) for prevalence when focusing on low risk of bias studies (refer to Table 4).

**Table 4:** Assessment of the risk of bias of included studies

Included study	Appropriate sampling frame	Using a proper Sampling technique	Adequate sample size	Adequate description of study subject and setting	Sufficient data analysis	Use of valid methods for the conditions	Valid measurement for all participants	Using appropriate statistical analysis	Adequate response rate	Overall quality (Rate over 9)
(Yılmaz and Coşkun 2019) [23]	1	0	0	0	1	1	1	0	0	4/9
(Kibar, Dalyan Aras et al. 2017) [25]	1	0	0	0	1	1	1	1	1	6/9
(Erdogan Iyigun, Selamoglu et al. 2015) [26]	1	1	0	0	0	1	1	0	1	5/9
(KİBar, Aras et al. 2015) [27]	1	0	0	0	1	1	1	0	1	5/9
(Ay, Kutun et al. 2014) [28]	0	1	1	1	1	1	1	0	1	7/9
(Ozcinar, Guler et al. 2012) [29]	1	1	0	0	1	1	1	1	0	6/9
(Ozaslan and Kuru 2004) [30]	1	1	0	0	1	1	1	1	1	7/9
(Rebegea, Firescu et al. 2015) [21]	1	1	0	0	1	1	1	1	1	7/9
(Borman, Yaman et al. 2018) [31]	1	0	0	0	0	1	1	1	0	4/9
(Vieira, Silva et al. 2018) [32]	1	0	0	0	0	1	1	0	0	3/9
(Borman, Yaman et al. 2017) [51]	1	1	0	0	1	1	1	0	0	5/9
(Godoy, Dias et al. 2014) [33]	0	1	1	0	0	0	1	0	1	4/9
(Paiva, Rodrigues et al. 2013) [34]	1	1	0	0	1	1	1	1	1	7/9
(do Nascimento, de Oliveira et al. 2012) [24]	0	1	1	0	0	0	1	0	1	4/9
(de Godoy, Barufi et al. 2012) [35]	0	0	0	0	0	1	1	0	0	2/9
(Campanholi, Duprat et al. 2011) [20]	0	0	0	0	0	1	1	0	1	3/9
(Bergmann, Bourrus et al. 2011) [36]	1	1	0	0	1	1	1	1	1	7/9
(Velloso, Barra et al. 2011) [37]	1	0	0	0	1	1	1	0	0	4/9
(Alem and Gurgel 2008) [38]	1	0	0	1	0	1	1	0	0	4/9
(Paim, Lima et al. 2008) [5]	0	1	1	0	1	1	1	0	1	6/9
(Batiston and Santiago 2005) [39]	0	0	0	0	1	0	1	1	0	3/9
(Elumelu-Kupoluyi, Adenipekun et al. 2013) [40]	0	1	0	0	0	1	1	0	0	3/9
(Khanna, Gupta et al. 2019) [41]	1	1	0	0	0	1	1	1	1	6/9
(Rastogi, Jain et al. 2018) [42]	1	1	0	0	1	1	1	0	1	6/9
(Gopal, Acharya et al. 2017) [43]	0	1	0	0	0	1	1	0	1	4/9
(Nandi, Mahata et al. 2014) [52]	1	0	0	0	0	1	0	0	1	3/9
(Raja, Damke et al. 2014) [44]	0	0	0	0	0	0	0	0	1	1/9
(Deo, Ray et al. 2004) [45]	1	1	0	0	1	1	1	0	1	6/9
(Halder, Morewya et al. 2001) [46]	0	1	1	1	0	0	0	0	1	4/9
(Haddad, Farzin et al. 2010) [54]	1	1	1	1	1	1	1	1	1	9/9
(Honarvar, Sayar et al. 2016) [47]	1	1	1	0	1	1	1	1	1	8/9
(Morcos, Al Ahmad et al. 2013) [48]	1	0	1	0	1	1	1	0	1	6/9
(de Melo Ferreira, de Figueiredo et al. 2012) [49]	1	0	0	0	1	1	0	1	1	5/9
(Eke, Alabi-Isama et al. 2010) [53]	0	0	0	1	0	0	0	0	1	2/9
(Marin, Pleşca et al. 2014) [50]	0	1	1	0	0	0	0	0	1	3/9
(Dem, Kasse et al. 2001) [22]	0	1	0	0	0	0	0	0	1	3/9

A post-hoc subgroup analysis was also conducted in which we removed from the meta-analyses all studies that had less than 24 months follow up (*n* = 5). This too resulted in minimal improvement in heterogeneity.

## Discussion

This systematic review and meta-analysis is the first to attempt to estimate prevalence and incidence of lymphedema in LMICs. Arm lymphedema results were too heterogeneous to reliably estimate prevalence or incidence. Two studies suggest that the prevalence of leg lymphedema may be between 7 and 13% [22, 50], while only one study estimated incidence of leg lymphedema, estimating it to be 59.1%, focusing specifically on Brazilian patients following ilioinguinal lymphadenectomy [20].

Differences in study quality, sample size estimations, technique of sampling and study methodology typically form the bases for heterogeneity in meta-analysis of prevalence or incidence data, and this review is likely to be no exception. Lymphedema following cancer treatment might be influenced by lymphatic drainage, adjuvant radiation therapy, hormonal therapy, skin care, physiotherapeutic modalities such as simple home exercises, and self-lymphatic drainage techniques [55] and trastuzumab therapy and taxane-based chemotherapy [56], but none of these variables were reliably reported. Risk factors for arm lymphedema following breast cancer treatment identified by this review did not differ from those identified by studies in HICs [6, 57, 58]. BMI ≥25, age above 60 years, having axillary radiotherapy treatment with axillary lymph node dissection, ≥16 lymph nodes removed, higher lymph node ratio, and increased engagement in moderate to severe physical activity were identified as the most significant risk factors of arm lymphedema. The number of lymph nodes typically removed in LMICs may be more compared to HICs because of later detection of cancer and differences in t the type of treatment provided as standard. Such differences in treatments and health management practices in LMICs are likely to have accounted for at least some of the variation found between the current review and that conducted in HICs [59]. Lymphedema incidence and prevalence were generally higher in our review compared to the previous review of studies conducted in HICs [6]. However, comparability between these reviews is limited by the heterogeneity among estimates and general low quality of studies in LMICs [6]. However, comparability between these reviews is limited by the heterogeneity among estimates and general low quality of studies in LMICs..

While several different methods are available for measuring lymphedema, the majority of studies included in this review used the circumferential measurements and patients’ self-reports. Circumferential measurement is a non-invasive, inexpensive and practical method of lymphedema measurement in the clinical setting [6, 60] with established reliability [61]. Self-report, on the other hand, is open to subjective variability between patients and is typically used in the clinic to assess the patient’s view of improvement [6, 60] and likely to report higher rates compared with the more objective lymphedema measurement methods like circumferential measurements [62]. One study [26] reported on the use of bioimpedance spectroscopy in diagnosing lymphedema. Although this method has demonstrated high sensitivity and specificity, the equipment is expensive and few health facilities even in HICs are able to afford it [63], prohibiting its use in LMICs.

### Limitations

The limitations of this study arise from the limited number of available studies and incomplete reporting, especially with regard to disease stage and treatment. Studies were limited to a small range of countries in certain geographical regions. None of the studies controlled for premorbid lymphedema.

### Implications for future research

Notable gaps that should be filled by future research include studies of the prevalence of lymphedema in certain geographical regions, such as Africa. Because affected people may sometimes resort to traditional and other alternative treatment rather than hospitals in the first instance [64], community-based research may be necessary. In the absence of a gold standard lymphoedema measurement, reaching global consensus on the most reliable and feasible method of identifying lymphedema in LMICs would do much to enable comparability between studies, and to assess the impact of any treatments. Understanding the impact the role of social-determinates of health and culture have on lymphedema prevalence and incidence rates in LMICs are important areas for future research.

Lymph node sparring is considered an invaluable surgical method for lymphedema prevention [65]. However, due to the quality of reporting we were unable to examine its impact on lymphedema prevalence or incidence in LMICs.

## Conclusion

This systematic review and meta-analysis was unable to reliably estimate the prevalence or incidence of lymphedema in LMICs due to heterogeneity (arm lymphedema) and small numbers of studies (leg lymphedema). Heterogeneity among estimates is likely due to differences in measurement methods, as well as variability in stage of cancer, treatments and other variables not reliably reported. Rates were higher according to self-report or compared with more objective measures, such as the clinical diagnosis or the circumferential measurements. Gaining consensus on how best to measure lymphedema in LMICs would enable comparability between studies and more reliable estimates. Better understanding the factors contributing to the wide variability in arm lymphedema is an important first step to developing targeted interventions to improve the quality of life of people living with cancer related lymphedema in LMICs.

## Data Availability

The datasets used and/or analysed during the current study are available from the corresponding author on reasonable request.

## Abbreviations

LMIC: Low and middle-income countries
EMBASE: Excerpta medica database
CINAHL: Cumulative Index of Nursing and Allied Health Literature
CI: Confidence interval
PROSPERO: Prospective Register of Systematic Reviews
PRISMA: Preferred reporting items for systematic reviews and meta-analyses
BMI: Body mass index
HIC: High-income countries
UK: United Kingdom
OR: Odds ratio
HR: Hazard ratio
